# When disaster strikes: staff recall and the use of staff recall systems during mass patient influx at Norwegian emergency primary health care centers – a cross-sectional study

**DOI:** 10.1186/s12873-023-00802-0

**Published:** 2023-03-13

**Authors:** Fredrik Femtehjell Friberg, Heléne Nilsson, Ann-Chatrin Linqvist Leonardsen

**Affiliations:** 1 Department of Anesthesiology and Intensive Care, Oslo University Hospital, Ullevål, NO-0450 Oslo, Norway; 2 Ostfold University College, NO-1757 Halden, Norway; 3 Department of Anesthesiology and Intensive Care, Ostfold Hospital Trust, NO-1714 Grålum, Norway; 4 The Swedish Civil Contingencies Agency, SE-65181 Karlstad, Sweden; 5 Faculty of Medicine and Health Science, University of Linköping, SE-58183 Linköping, Sweden

**Keywords:** Automatized staff recall system, Mass patient influx, Mass casualty incident, Disaster plan, Staff recall, Mass notification system, Questionnaire

## Abstract

**Background:**

In Norway, planning for disasters has been specifically emphasized since the incidents on July 22^nd,^ 2011. Every municipality is now legislated to have a contingency plan that includes plans for staff recall during situations with mass influx of patients. Whether the primary health care services in Norway are prepared for mass influx of patients remains unclear.

**Aims of the study:**

The aims of this study were (1) to assess the experiences of head doctors at emergency primary health care centers (EPHCC) in Norway with mass influx of patients, (2) to explore mass influx and staff recall procedures in use, (3) to assess head doctors’ experiences with staff recall systems, and (4) to assess their perspective on automatized staff recall systems. We also wanted to assess whether there were differences between small and large EPHCCs regarding whether they had plans in place.

**Methods:**

The study had a cross-sectional, multicenter design, using a self-developed questionnaire. The questionnaire was developed utilizing recommendations from the Delphi technique, including an expert group and piloting. A purposive sampling strategy was used, including head doctors from Norwegian EPHCCs (n = 169). Data were analyzed using the Statistical Package for the Social Sciences, and included descriptive statistics, Chi-Square tests and Shapiro-Wilks. Free-text answers were analyzed by content analysis.

**Results:**

A total of 64 head doctors responded to the questionnaire. The results show that 25% of the head doctors had experienced mass influx of patients at their EPHCC. In total 54.7% of Norwegian EPHCCs did not have disaster plans that consider mass influx situations. The majority of EPHCCs plan to recall staff one by one (60.3%) or through Short-Message-Systems (34.4%). Most EPHCCs had available telephone “alarm” lists (81.4%), that are updated regularly (60.9%). However, only 17.2% had plans that consider loss of mobile phone connection or internet. In total, 67,2% of the head doctors reported to have little experience with automatized staff recall systems, and 59,7% reported to have little knowledge about such systems. There were no significant difference between small and large EPHCCs in having plans or not.

**Conclusion:**

Even though our results show that few EPHCCs experience mass influx of patients, it is important to be prepared when such incidents do occur. Our results indicate that it is still potential for improvement regarding plans for staff recall and implementation of staff recall systems at Norwegian EPHCCs. Involving national disaster medicine experts in the process of generating tools or checklists could aid when constructing disaster plans. Education and implementation of training for mass influx situations at all levels should always be highlighted.

## Background

In Norway, healthcare related contingency and crisis planning became a major concern primarily during and after the 2nd World War. The war illustrated the evident need for disaster planning, resulting in the establishment of councils and boards focusing on disaster preparedness and mitigation in the healthcare sector [1]. The first Act on health and social emergency preparedness was approved in 1955, but has since been subjected to numerous revisions, latest in July 2001 [2]. A municipal contingency preparedness handbook, with a chapter dedicated to health-related emergency and contingency preparedness, was published in 1989 [3]. However, from the perspective of mass casualty incidents, the modern Norwegian timeline dates pre and post July 22nd, 2011. On this date, a sole right-wing extremist carried out two more or less simultaneous attacks, striking both the headquarter of politics in Oslo, and the socialist summer youth camp on Utøya, an island about 40 km North-West of Oslo. The attacks led to the death of 77 persons, mostly children, and hundreds of injured. The elicited emergency response to Utøya and Oslo was unparalleled in Norway, demanding an enormous cross-agency collaboration, including municipal, county and governmental health actors [4]. The health response was of high quality, as both concluded by the July 22nd Commission and the Norwegian Directorate for Health [5, 6]. However, the International Advisory Council that contributed to the latter report underlined the lack of a national disaster preparedness plan, and a unified national triage system for mass casualty incidents. The prehospital emergency medicine community in Norway had expressed the need for a national interagency standard for disaster triage a year prior to the event [7].

What Norway experienced post 22nd of July 2011 in terms of preparations for future disasters are relatable to what superseded the terrorist attacks on September 11 in New York, the London bombing in 2005, and the Paris attacks in 2015 [8–10]. A national guideline for mass casualty incident triage were established in 2013 [11], adapting the “Sort, Assess, Lifesaving interventions, Treatment/Transport” (SALT) system [12]. The Norwegian Emergency Medicine Regulation, revised in 2015, states that every emergency primary health care center (EPHCC) must have a doctor on call 24/7, with a defined skillset and experience [13]. Moreover, both the Act on contingency planning in healthcare and the National guideline for EPHCCs state that every municipality must have a contingency plan that includes plans for staff recall during situations with mass influx of patients [2, 14, 15]. Additionally, the National guideline for mass casualty incident site organization, published in 2020, states that EPHCCs must have tools and guidelines for recalling staff in the case of exhausted staff resources [16]. Legislations on elements of municipal contingency and crisis planning were implemented as late as in 2021 [17]. Most of the literature on disaster response in Norway analyze hospitals [18]. Little research has been conducted on the role of primary health care in disaster and emergency medicine [19, 20]. When scoping international literature, Mathew and Hubloue recently published a study on mass casualty incident readiness at primary health care centers in Qatar, prior to the 2022 FIFA World Cup [21]. Here, the conclusion was that plans for mass casualty incidents do not exist. Despite the increasing focus on disaster planning in Norwegian health care, a national, or regional coordinating plans for cross-agency collaboration during mass casualty incidents do not exist. Neither does a disaster plan that involves coordination between governmental and primary health care resources.

The World Health Organization (WHO) defines mass casualty incident as “events” that generate more patients at one time, than locally available resources can manage using routine procedures [22]. Surge capacity is defined by the American College of Emergency Physicians as a measurable representation of a healthcare system’s ability to manage a sudden influx of patients, considering variables such as staff, supplies, structure, system and space [23]. When considering human resources during mass influx of patients, a consistent feature is that help will commonly be offered in abundance from bystanders or volunteers [24]. However, being able to get the right people, at the right time, to the right place, needs coordination and requires specific focus when planning for disasters. Staff recall to meet disasters or emergency situations was described in the literature 50 years ago [25]. At that time, staff recall depended on pyramid warden systems and telephone trees. Since then, to our knowledge, few studies have been conducted on staff recall in the context of mass casualty incidents. Moreover, these few studies focus on hospital emergency departments, and the use of telephone trees, short-message-systems (SMS), and application-based recall [26–28]. Most hospitals in Norway use a mass notification system, in this study called automatized staff recall system [29], like Everbridge or HelseCIM. These systems are designed to recall staff on a predefined list upon activation, delivering a voice message to rapidly assess available personnel. The same system can initiate automatized recalling of staff, saving crucial time and staff resources that otherwise would be occupied with recall. Moreover, most of Norwegian hospitals have detailed plans on how to respond to mass influx of patients, but whether the emergency primary health care providers in Norway are prepared to the same extent remains unclear.

## Methods

### Aims

The aims of this study were (1) to assess the experiences of head doctors at EPHCCs in Norway with mass influx of patients, (2) to explore mass influx and staff recall procedures in use, (3) to assess head doctors’ experiences with staff recall systems, (4) to assess head doctors’ perspectives on automatized staff recall systems, and (5) to assess whether there were differences between small and large EPHCCs regarding whether they had plans in place.

### Design

The study had a cross-sectional, multicenter design, using a self-developed questionnaire. The study adheres to the STROBE guidelines [30].

### Development of the questionnaire

We were not able to identify relevant or relatable studies, or any questionnaires designed to assess the use of staff recall or staff recall systems during mass patient influx. Hence, recommendations from the Delphi technique were used to develop a questionnaire [31]. This technique is used to obtain expert opinions systematically and consist of three steps: 1) expert inputs, 2) interaction with feedback, and 3) statistical group responses. Confidentiality is highlighted as an important part of the process [32]. In this study, the first two steps were utilized. Experts were defined as “informed individuals”, “specialists in their field”, and professionals that had expertise in either disaster planning, staff recall, staff recall systems, EPHCC disaster preparedness, or questionnaire development. The expert group (n = 12, of which 7 female) included eight anesthesiologists, two EPHCC leaders, one district municipal head doctor, and one professor and expert on questionnaire development. After several rounds of inputs and revising, a consensus was reached, and questions and answering alternatives were adjusted accordingly. The questionnaire consisted of 50 questions. Response alternatives were either “yes”, “no”, “not sure”/”do not know” or on a five-point Likert scale, where 1 = to a very low degree, 2 = to a low degree, 3 = neither/nor, 4 = to a high degree, and 5 = to a very high degree. Additionally, there were four free-text questions.

### Pilot study

After the first step in the development process, the questionnaire was piloted to control for face and content validity on 15 general practitioners (n = 15, of which 11 male), also working in an EPHCC, with a nationwide distribution. The general practitioners were asked to report consistently in a form, whereas the questions were relevant and not leading, clear, logical, if the questions could be misunderstood, and the time spent to complete the questionnaire. Answers were recorded, and the final alterations were made on the questionnaire before initiation of the survey.

### Setting

Norway has a total of 5,475 million inhabitants, spread over 323 810 square kilometers [33, 34]. Consequently, all inhabitants in Norway does not have access to the same quality of health services, especially when in need of emergency trauma care. There are 34 hospitals with emergency surgery capabilities, six of these are regional trauma centers/University hospitals [35]. Since hospitals are spread thin across the country, the districts are equipped with municipal or intermunicipal EPHCCs to provide emergency medical care. Norway has a total of 169 EPHCCs, located across the country, staffed by general practitioners and nurses. Some of the EPHCCs cover cities and several hundreds of thousand inhabitants, while others serve only a few hundred inhabitants while covering a rather large geographical area [36]. In the case of a mass casualty incident in Norwegian districts, the capacity at most EPHCCs will most definitely be challenged, and to a varying degree rapidly exhausted.

### Sample

We used a purposive sampling strategy, aiming at including one participant from all Norwegian EPHCCs. Hence, no sample size calculations were performed. The inclusion criterium was that participants should have a position as a head doctor at an EPHCC. The decision to include only head doctors and not EPHCC leaders were made to be able to include more of the smaller EPHCCs that do not have an administration. Since there is not an official list of email addresses to Norwegian EPHCC head doctors, a considerable effort was made to collect contact information to all 169 EPHCCs in Norway. All requests were made via the various municipalities’ administrations; however, the response was varying. Several EPHCCs responded that they were too small and did not have a head doctor, some were not organized with an administrative structure. What we learned from this is that the number of EPHCC head doctors in Norway does not comply with the number of EPHCCs. After 2 months of trying to collect email addresses and contact information, a total of 66 email addresses were achieved. An additional 28 emails were sent to municipal head doctors that also serves as the district EPHCC head doctor. The remaining EPHCCs, to which we were not able to retrieve digital contact information, received a written letter with information about the study and an invitation to participate.

### Data Collection

Data collections was handled with Nettskjema.no, a survey solution developed and hosted by the University of Oslo.

The first invitation to participate in the study was sent 20th of September, 2022. Two reminders were sent, one on the 28th of September, the last on the 24th of October. The questionnaire was closed for submitting answers on the 14th of November, 2022.

### Analysis

Data were transferred from Nettskjema via Microsoft Excel to the Statistical Package for the Social Sciences, version 29.0 [37]. Descriptive statistics and frequenzies were used to analyze the data. The results are presented as median (Interquartile Range, IQR) or as number of respondents (n) and percentages as appropriate. Correlations were tested with Chi-Square test. Internal consistency of the questionnaire was tested by Cronbach’s alpha. Likert questions were tested for normality using Shapiro-Wilks test. The free text responses were analyzed through a simple content analysis, reading through responses, and searching for similarities and code-word repeat [38].

## Results

### Reliability and validity of the questionnaire

The Cronbach’s alpha was 0.69, which is assumed acceptable [39]. Moreover, results from the pilot study showed that the questionnaire was clear, logic and relevant, supporting the face and content validity of the questionnaire.

### Respondents

A total of 64 EPHCC head doctors (37.9%) responded to the questionnaire on behalf of their EPHCC. These counted for 13 (out of 54 = 24.5%) of EPHCCs in the Northern Norway, 6 (out of 24 = 24%) in Central Norway, 18 (out of 35 = 51,4%) in the Western Norway, and 27 (out of 52 = 51.9%) in the South-Eastern Norway Regional Health Authorities.

The characteristics of the EPHCCs represented are summarized in Table 1.

**Table 1:** Characteristics of the EPHCCs represented (n = 64)

		Median (IQR)	
Number of medical doctors present at EPHCC in a day (24/7)		2 (2)	
Number of nurses present at EPHCC in a day (24/7)		4 (6)	
			n (%)
Doctors on call at home	Yes	36 (59)	
	No	25 (41)	
Call out time for doctors at home (For EPHCC’s that have this arrangement)	< 30 min	22 (61.1)	
	31–60 min	13 (36.1)	
	61–90 min	1 (2.8)	
	> 90 min	-	
Distance from EPHCC to nearest referral hospital	< 50 km	44 (68.8)	
	51–100 km	11 (17.2)	
	101–150 km	4 (6.3)	
	151–200 km	1 (1.6)	
	> 200 km	4 (6.3)	
Type of nearest referral hospital (35)	Regional trauma center/University hospital	16 (25)	
	Large emergency hospital	27 (42.2)	
	Emergency hospital	20 (31.3)	
	Non-emergency hospital	1 (1.6)	

EPHCC = Emergency primary health care center. IQR = Interquartile range

Table 1 shows that most of the EPHCCs had less than 30 min call-out time for doctors at home, and were located < 50 km from the nearest referral hospital.

### Experiences with mass influx of patients

Among respondents, 25% answered that they had previous experience with mass influx of patients at their EPHCC. Additionally, 20% answered that they had experienced a mass patient influx that exhausted their staff resources in such a way that plans for staff recall were executed.

### Plans for mass influx of patients

Table 2 presents the results regarding the EPHCCs plan for mass influx of patients.

**Table 2:** Mass casualty incident plans at EPHCCs (n = 64)

		n (%)
EPHCCs has MCI plans	Yes	28 (43.8)
	No	35 (54.7)
	Do not know	1 (1.6)
EPHCC MCI plan consider staff recall (For the EPHCCs that have MCI plans)	Yes	25 (39,1)
	No	13 (20,3)
	Do not know	0 (-)
Plans easily accessible for EPHCC on-call staff	Yes	45 (70.3)
	No	12 (18.8)
	Do not know	6 (9.4)
EPHCC encourages staff to familiarize with the plan	Yes	29 (45.3)
	No	27 (42.2)
	I do not know	7 (10.9)
Plan is reviewed with staff on education days	Yes	13 (20.3)
	No	42 (65.6)
	I do not know	3 (4.7)
	Not relevant	6 (9.4)
Standardized forms for documenting MCI details at EPHCC	Yes	12 (18.8)
	No	44 (68.8)
	Do not know	8 (12.5)
Having different levels of activation at EPHCC	Yes	12 (18.8)
	No	50 (78.1)
	Do not know	2 (3.1)
Coordination of mass influx situation with hospitals, other EPHCC, and/or incident command	Direct communication	19 (29.7)
	Through emergency dispatch central	26 (40.6)
	No plans or procedures	19 (29.7)

EPHCC = Emergency primary health care center. MCI = Mass casualty incident

As many as 54,7% of the head doctors replied that their EPHCC did not have plans for handling mass influx of patients. Additionally, 68,8% reported that the EPHCC did not have standardized forms for documenting MCI details, and 29,7% did not have any plans for coordination of mass influx situations with other organizations.

When asked about available tools and checklists, developed by national experts, that may guide and aid in the development of MCI plans at the EPHCC, 4.8% answered that the existing tools and checklists were “satisfactory”, 27.0% answered that the existing material was “not satisfactory”, and as many as 68% answered that they “do not know”. When asked if the plan would be more well prepared and comprehensive if they had national tools or checklist to aid them in planning, 62.5% answered “yes”, 6.3% answered “no”, and 31.3% answered that they “do not know”.

### Plans for staff recall during mass influx of patients

Table 3 presents a summary of how EPHCCs plan to recall staff during mass influx of patients can be found in Table 3.

**Table 3:** Staff recall during mass influx of patients at Norwegian EPHCCs (n = 64)

		n (%)
How EPHCCs recall staff during mass influx	Call all staff one-by-one	38 (60.3)
	SMS all staff	22 (34.4)
	Call all staff at once	2 (3.2)
	Automatized staff recall system	1 (1.6)
Responsible for recalling staff during mass influx of patients	On-call nurse	29 (45.3)
	Leader (at home or present)	19 (29.7)
	On-call doctor	8 (12.5)
	Other personnel / Do not know	7 (11.0)
Have telephone (“alarm”) lists accessible for on-call personnel at EPHCC	Yes	52 (81.4)
	No	11 (17.2)
	Do not know	1 (1.6)
Updates telephone (“alarm”) lists regularly (Every 3rd month or when new staff are employed)	Yes	39 (60.9)
	No	14 (21.9)
	I do not know	5 (7.8)
	Not relevant	6 (9.5)
Plans that take loss of internet or mobile phone connection into consideration	Yes	11 (17.2)
	No	43 (67.2)
	Do not know	7 (10.9)
	Not relevant	3 (4.7)
Memorandums of Understanding to increase staff surge capacity with other health care actors in district	Yes	25 (39.1)
	No	36 (56.3)
	I do not know	3 (4.7)
Need for other non-emergency medical professionals (security, psychosocial services, funeral service, cleaning personnel, etc.) during a mass influx situation at the EPHCC	Yes	47 (73.4)
	No	8 (12.5)
	Do not know	9 (14.1)
Contact information to non-emergency medical services in plans	Yes	30 (46.9)
	No	22 (34.4)
	Do not know	3 (4.7)
Contact information to non-emergency medical services and other district health care actors included in telephone (“alarm”) lists, available for on-call personnel at EPHCC	Yes	37 (57.8)
	No	9 (14.1)
	Do not know	12 (18.8)
	Not relevant	6 (9.4)

EPHCC = Emergency primary health care center, SMS = Short-message-system

Table 3 shows that the most of respondents answered that they call all staff one-by-one (60,3%) during mass influx of patients to the EPHCC. Additionally, 46.9% answered that they have both EPHCC and emergency phone operators located in the same place, 20.3% answered EPHCC, 18.8% answered emergency phone operators, and 14.1% answered that they do not know where the responsibility lies.

In a free-text question, head doctors were asked to list any systems used for recall. We got 19 (n = 19) replies, 45 (n = 45) did not answer the question. Legevakt.no (n = 4) and GAT (n = 3) were the most used systems. Both systems send an SMS to a predefined group. One respondent answered that the EPHCC had a plan that included a telephone-tree system. One respondent answered that the EPHCC utilize an automatized staff recall system, in this case Everbridge♥. Another respondent answered that EPHCC had helseCIM♥ installed, but that it was not operational.

### Head doctors’ experiences with staff recall systems in use

Table 4 gives an overview of head doctors’ experience with the staff recall procedure and/or systems in use at their EPHCC.

**Table 4:** Head doctor’s experience with staff recall and automatized staff recall systems (N = 54)

	Low degree	Neither/Nor	High degree
Head doctors knows EPHCC plans for recalling staff during mass influx of patients	22.2%	11.1%	66.6%
Head doctors know when to execute plans for staff recall	19%	22.2%	58.8%
EPHCC staff are trained in executing plans for staff recall	42.9%	20.6%	36.5%
EPHCC will execute plans for staff recall when expecting 5 patients	37.5%	17.2%	45.4%
EPHCC will execute plans for staff recall when expecting 10 patients	21.8%	3.1%	75%
EPHCC will execute plans for staff recall when expecting 15 patients	17.2%	0%	82.8%
The system used for recalling staff now is well-functioning	35.9%	35.9%	28.1%
Head doctor’s experienced with use of automatized staff recall systems (ex. previous job in hospital)	67.2%	7.8%	25%
Have knowledge with automatized staff recall systems	59.7%	17.7%	22.6%
Consider plan to call all staff on-by-one as good enough	50%	21.0%	29%
Consider plan to send an SMS to all staff as good enough	48.5%	25%	26.6%
Believe that an automatized staff recall system would streamline the process of recalling staff at the EPHCC	25%	28.3%	46.7%
Believe that an automatized staff recall system would relieve the staff at the EPHCC, to perform other tasks	15%	28.3%	56.7%
Need for a better staff recall system at the EPHCC	25%	39.1%	35.9%

EPHCC = Emergency primary health care center. Scored on a Likert scale, where 1 = to a very low degree, 2 = to a low degree (1 + 2 recoded to 1 = low degree), 3 = neither/nor (recoded to 2 = neither/nor), 4 = to a high degree, and 5 = to a very high degree (4 + 5 recoded to 3 = high degree).

### Head doctors’ perspective on automatized staff recall systems

Table 5 gives an overview of head doctors’ perspective on automatized staff recall systems.

**Table 5:** Head doctors’ perspective on automatized staff recall systems (N = 54)

	Low degree	Neither/Nor	High degree
Head doctor’s experienced with use of automatized staff recall systems (ex. previous job in hospital)	67.2%	7.8%	25%
Have knowledge with automatized staff recall systems	59.7%	17.7%	22.6%
Believe that an automatized staff recall system would streamline the process of recalling staff at the EPHCC	25%	28.3%	46.7%
Believe that an automatized staff recall system would relieve the staff at the EPHCC, to perform other tasks	15%	28.3%	56.7%

EPHCC = Emergency primary health care center. Scored on a Likert scale, where 1 = to a very low degree, 2 = to a low degree (1 + 2 recoded to 1 = low degree), 3 = neither/nor (recoded to 2 = neither/nor), 4 = to a high degree, and 5 = to a very high degree (4 + 5 recoded to 3 = high degree).

Table 5 shows that 67,2% of the head doctors had little experience with automatized staff recall systems, and 59,7% had little knowledge about such systems. The head doctors believed that automatized staff recall systems to a high degree would streamline the process (46,7%) and relieve staff at the EPHCC (56,7%).

When asked about the technical aspects of using an automatized staff recall system, only 1,6% answered that they believe such a system to be technically difficult. Also, 34.4% answered that such a system was not technically difficult, while 64% answered either that they did not have any experience with automatized staff recall, or that they do not know. When asked about the cost, 15.9% answered that such a system comes with a considerable price. 9.5% answered that they do not think that the system was expensive, and a total of 74.6% answered either that they do not have any experience with such a system or that they do not know.

### Differences between small and large EPHCCs?

To assess whether there were differences in having mass casualty plans or not between large and small EPHCCs, we ran a Chi-square test between EPHCCs with three doctors or less and EPHCCs with more than three doctors, and whether they had mass casualty incident plans. We found a non-significant difference, Chi-square = 0.998 (p = 0.05).

## Discussion

Our result show that 25% of EPHCC head doctors had experienced mass influx of patients at their EPHCC. In total, 54.7% of Norwegian EPHCCs do not have disaster plans that consider mass influx situations. The majority of EPHCCs plan to recall staff one by one (60.3%) or send SMS (34.4%), which were both viewed by most of the head doctors as insufficient. Most of the EPHCC head doctors responded that they have little experience with and knowledge about automatized staff recall systems. There was no significant difference regarding having an MCI plan in small or large EPHCCs.

In our study, 25% responded that they had previous experience with mass influx of patients and 20% answered that they had experienced a mass patient influx that exhausted their staff resources in such a way that plans for staff recall were executed. In 2019, the Norwegian Directorate for Civil Protection and Emergency Planning published a hazard-vulnerability analysis with potential disaster scenarios in Norway [40]. In this analysis, out of the ten scenarios with high to very high probability, several would result in a potential MCI that would require executing disaster plans at Norwegian EPHCCs. From an international perspective, primary health care plays an important role in disaster response. During the hurricane Katrina in 2005, a primary health care facility provided medical care to 1664 patients during a 2-week period [41]. From a Norwegian perspective, EPHCCs have played a major role in the emergency medical treatment chain for many years [42]. In certain settings, the EPHCC might be the only alternative for inhabitants in remote areas, thus underlining the need for a plan should a disaster occur.

However, in our study, more than half of the respondents replied that they do not have plans for handling mass influx of patients. We assumed that smaller EPHCCs might not be as prepared to handle mass influx situations as larger EPHCCs. However, when analyzing for this hypothesis, there was not a significant relationship between the size of the EPHCC and if they have mass casualty incident plans (Chi-Square = 0.998, p = 0.05). By introducing general concepts in disaster planning, a clear contingency plan can minimize unnecessary problems during a disaster and help save more lives [43]. WHO published the Health Emergency and Disaster Risk Management (H-EDRM) Framework in 2019, emphasizing the need for disaster preparedness in all sectors of the health system, including primary health care [44]. Disaster plans that consider mass influx of patients are also encouraged by Norwegian laws and regulation [14, 17, 45].

When asked whether national developed tools or checklists were available to aid EPHCCs in making more uniform and comprehensive disaster plans, 4.8% answered that the existing material were satisfactory. Most of the respondents agreed that their plans for mass influx of patients would have been better and more comprehensive, if prepared while utilizing specifically constructed tools or checklists developed by disaster medicine experts. James et al. [46], indicate that proficiency in disaster medicine and public health preparedness requires knowledge and skills beyond those typically acquired in clinical and public health training and practice. This underlines the need for expert involvement in the process of constructing disaster plans.

In our study, most of the head doctors replied that non-medical professionals are required in their EPHCCs during mass influx situations. Considerations regarding what kind of non-medical professionals required during a situation with mass influx of patients are well described in the literature, and can be found in the functional annexes made by the United States Federal Emergency Management Agency (FEMA), comprehensive preparedness guide (CPG) 101 [47]. When asked about agreements to increase surge capacity with staff not normally employed at the EPHCC, 39,1% replied to have made such arrangements with other health actors in their district. This is also in-line with findings from Dale et al., where 44.6% of the staff that contributed during the initial phase of the Covid-19 pandemic at Norwegian EPHCCs were other personnel than those employed at the EPHCC [48].

Only 39.1% of the EPHCCs responded that their MCI plans included how to recall staff. The on-call nurse was responsible for recall in most EPHCCs. When asked to assess how well they know their own plans for staff recall, 66,6% of head doctors replied that they know the plans well. However, when asked whether the staff are trained in executing the plans, only 36.5% of head doctors answered that they believed the staff to be well trained. Considerations on staff recall in disaster plans are mentioned as key components in both the chapter on surge capacity and the chapter on human resources in the hospital emergency response checklist [49], developed by WHO to assist hospital administrators and emergency managers in responding effectively to the most likely disaster scenarios.

On the question whether head doctors know when plans for staff recall should be activated in their own EPHCC, more than half of the head doctors responded that they knew the indication well. To elaborate this further, we asked whether their EPHCCs would need to activate staff recall if 5, 10 or 15 patients respectively were to arrive. To this, 45.5% of head doctors answered “to a high degree” for 5 patients, 75% answered “to a high degree” for 10 patients, while 82.8% answered “to a high degree” for 15 patients. A Finnish study from 2005 [50], with relatable epidemiology to Norway, found that the average number of patients in Finnish MCIs, with the majority being vehicle road accidents, were 4.5. Hence, more staff resources would be necessary in most EPHCCs, should a mass influx situation occur.

Plans for staff recall are kept easily accessible for on-call personnel in most EPHCCs. When asked whether the staff are encouraged to familiarize with the plans, 45.3% of the respondents answered that they encourage this. However, when asked about exercising plans for mass influx of patients on education days, 65.6% answered that they do not prioritize this. This correlates with the findings of the previously mentioned study by Dale et al. [48], who explored the preparedness and management during the first three months of Covid-19 from the aspect of Norwegian EPHCCs. Here, 66.7% responded that they had a pandemic plan, but only 4.2% had performed training for pandemic preparedness. Hence, it seems that EPHCCs have potential for being more prepared in regard of planning for disaster.

Most of the EPHCCs in our study responded that their recall plan is to call all staff one-by-one (59.4%). Most EPHCCs have telephone “alarm” lists accessible for on-call personnel, and the majority update the lists regularly. However, only 17.2% have plans that include loss of mobile phone and internet connection. An important reference that highlights the need for backup plans in case there is a loss of conventional means of communications, was during the hurricane Katerina in the United States. Here, emergency medical providers experienced the consequences of lost mobile phone and internet connection, having to rely on short wave radios and alternative means of communication [51]. Moreover, when head doctors were asked whether calling all staff one by one or sending SMS, is a good enough system, the majority agree that this is not an optimal system. The viability of using SMS or phone calling for staff recall, might be compromised during disasters. During routine network traffic, an analysis from 2007 shows that an SMS delivery failure rate of 5.1% can be expected [52]. During peaks of network traffic, a higher rate is seen with prolonged transmission delays for calls and a high frequency of dropped SMS. These finding question the sole use of mobile phone connection and internet for staff recall, should a disaster either directly cause connection failure or indirectly by overwhelming the local network.

Only 1.6% of the respondents replied that their EPHCC use an automatized staff recall system. However, about half of the respondents would expect an automatized staff recall system to streamline staff recall and relieve staff to perform other tasks, should recalling be necessary. Implications for a potential future use of such a system in Norwegian EPHCCs would require educating staff in automatized recall systems, then evaluate benefits and disadvantages, before evaluating if such a system could improve staff recall at Norwegian EPHCCs.

### Strengths and limitations

The respondents represented 64 out of 169 EPHCCs, hence the generalizability of our findings may be questioned. However, both the Western and South-East Norway Regional Health Authorities comprise about 80% of the population in Norway, in which we had a response rate of 51.4% and 51.9% respectively, which is assumed acceptable in questionnaire studies. Hence, we assume that our findings are transferable to the Norwegian setting.

Moreover, we used a self-developed questionnaire for data collection. However, we used a delphi-method approach, including experts and piloting, supporting the reliability and face and content validity of the questionnaire. Additionally, the Cronbach’s alpha was acceptable. In retrospect, we see that we should have included more questions concerning the relationship between the EPHCC and the emergency medical communications central. More questions regarding activation of voluntary organizations at local, municipal, or district level should also have been included. Unfortunately, this was not done.

It may be argued that a qualitative approach would be more appropriate when exploring participants’ experiences and perspectives. However, our aim was to explore this in a broad sample to get an overview of the Norwegian status regarding plans for MCI and staff recall.

## Conclusion

This study shows that Norwegian EPHCCs experience mass influx of patients, even if this fortunately seldom occur. Most EPHCCs would be in need for additional staff resources in such cases. The results from this study indicate that there is still potential for improvement regarding development and utilization of mass casualty incident and staff recall plans at Norwegian EPHCCs. Most of the EPHCC head doctors perceived that an involvement of national disaster medicine experts in the process of generating tools or checklists that could aid in constructing disaster plans, would be desired. Education and implementation of training for mass influx situations should always be highlighted.

### Implications for further research

There is little knowledge about automatized staff recall systems among Norwegian EPHCC head doctors, and whether such systems are a solution that can solve how EPHCCs recall staff for mass influx situations in the future require more research. Whether one of the systems listed in this study is better for staff recall requires more research. To explore aspects of staff recall systems assumed optimal by head doctors in EPHCCs, it could also be interesting to conduct a qualitative study.

Further research should also be focused on how we best can facilitate better planning and development of mass casualty incident plans for EPHCCs.

## Data Availability

Anonymous data is available upon request to the last author, Ann-Chatrin Linqvist Leonardsen: ann.c.leonardsen(at)hiof.no.

## List of abbreviations

EPHCC: Emergency primary health care centers
FEMA: Federal Emergency Management Agency
H-EDRM: Health Emergency and Disaster Risk Management
IQR: Interquartile range
MCI: Mass casualty incident
SALT: Sort, Assess, Lifesaving interventions, Treatment/Therapy
SMS: Short-message-system
WHO: World Health Organization

## References

[1] Odelstingsmelding nummer 79 (1999–2000). Innstilling fra sosialkomiteen om lov om helsemessig og sosial beredskap: Sosialkomiteen; 2000 [cited 2023 21.02]. Odeltingsproposisjon nr. 89]. Available from: https://www.stortinget.no/globalassets/pdf/innstillinger/odelstinget/1999-2000/inno-199900-079.pdf.

[2] Lov om kommunal helse. og omsorgstjeneste (Act on municipal health and care services), LOV-2011-06-24-30: Helse og omsorgsdepartementet (Department of Health); 2012 [cited 2022 08.12]. Available from: https://lovdata.no/dokument/NL/lov/2011-06-24-30#KAPITTEL_5.

[3] R BT. Beredskapshåndbok for kommunen [Electronic]. Direktoratet for sivilt beredskap 1989 [cited 2023 21.02]. Available from: https://www.nb.no/items/7d41d92f7552097786ac42571fccbf99?page=9.

[4] Sollid SJ, Rimstad R, Rehn M, Nakstad AR, Tomlinson A-E, Strand T (2012). Oslo government district bombing and Utøya island shooting July 22, 2011: the immediate prehospital emergency medical service response. Scand J Trauma Resusc Emerg Med.

[5] Norges offentlige utredning (NOU) 2012:14. Rapport fra 22. juli-kommisjonen (Report from the 22nd of July Commission) Oslo: 22. juli kommisjonen (22nd of July Commission). ; 2012 [cited 2022 08.12]. Available from: https://www.regjeringen.no/contentassets/bb3dc76229c64735b4f6eb4dbfcdbfe8/no/pdfs/nou201220120014000dddpdfs.pdf.

[6] Læring for bedre beredskap (Learning for better planning). Helseinnsatsen etter terrorhendelsene 22.juli 2021: Helsedirektoratet, Beredskapsavdelingen (Directorate of Health); 2011 [cited 2022 08.12]. Available from: https://www.helsedirektoratet.no/rapporter/laering-for-bedre-beredskap-helseinnsatsen-etter-terrorhendelsene-22.juli-2011/L%C3%A6ring%20for%20bedre%20beredskap%20-%20Helseinnsatsen%20etter%20terrorhendelsene%2022.%20juli%202011.pdf.

[7] Rehn M, Lossius H (2010). Katastrofetriage - behov for en norsk standard. Tidsskr Nor Laegeforen.

[8] Moran CG, Webb C, Brohi K, Smith M, Willett K (2017). Lessons in planning from mass casualty events in UK. BMJ.

[9] Hirsch M, Carli P, Nizard R, Riou B, Baroudjian B, Baubet T (2015). The medical response to multisite terrorist attacks in Paris. Lancet.

[10] Chartoff SE, Kropp AM, Roman P. Disaster Planning. StatPearls. Treasure Island (FL): StatPearls Publishing. Copyright © 2022. StatPearls Publishing LLC.; 2022.

[11] Norsk veileder for masseskadetriage (National guidelines for mass casualty incident triage): Helsedirektoratet (Directorate of Health). ; 2013 Available from: https://www.helsedirektoratet.no/veiledere/masseskadetriage/Masseskadetriage%20%E2%80%93%20Nasjonal%20veileder.pdf.

[12] Lerner EB, Schwartz RB, Coule PL, Weinstein ES, Cone DC, Hunt RC (2008). Mass casualty triage: an evaluation of the data and development of a proposed national guideline. Disaster Med Public Health Prep.

[13] Akuttmedisinforskriften (Emergency medicine regulation), FOR-2015-03-20-231: Helsedirektoratet (Department of Health). ; 2015 [cited 2022 08.12]. Available from: https://lovdata.no/dokument/SF/forskrift/2015-03-20-231#KAPITTEL_2.

[14] Helseberedskapsloven (Act on contingencyplanning in healthcare)., LOV-2000-06-23-56: Helsedepartementet (Department of Health); 2001 [cited 2022 08.12]. Available from: https://lovdata.no/dokument/NL/lov/2000-06-23-56.

[15] Williams J, Freeman CL, Goldstein S. EMS Incident Command System. StatPearls. Treasure Island (FL): StatPearls Publishing. Copyright © 2022. StatPearls Publishing LLC.; 2022.

[16] Nasjonal veileder for helsetjenestens organisering på skadested. (National guidelines for mass casualty incident site organization) [Electronic]. Helsedirektoratet (Directorate of Health); 2020 [Available from: https://www.helsedirektoratet.no/veiledere/helsetjenestens-organisering-pa-skadested/Helsetjenestens%20organisering%20p%C3%A5%20skadested%20%E2%80%93%20Nasjonal%20veileder.pdf.

[17] Veileder til forskrift om kommunal beredskapsplikt (Regulation on municipal contingency planning). : Direktoratet for samfunnsikkerhet og beredskap (Directorate for Civil Protection and Emergency Planning); 2021 [cited 2022 08.12]. Available from: https://www.dsbinfo.no/DSBno/2021/veiledning/veileder-til-forskrift-om-kommunal-beredskapsplikt-versjon-2-september-2021/.

[18] Gaarder C, Jorgensen J, Kolstadbraaten KM, Isaksen KS, Skattum J, Rimstad R (2012). The twin terrorist attacks in Norway on July 22, 2011: the trauma center response. J Trauma Acute Care Surg.

[19] Zakariassen E, Burman RA, Hunskaar S (2010). The epidemiology of medical emergency contacts outside hospitals in Norway - a prospective population based study. Scand J Trauma Resusc Emerg Med.

[20] Rørtveit S, Hunskår S (2009). [Medical emergencies in a rural community]. Tidsskr Nor Laegeforen.

[21] D M, I H. The Readiness of Primary Healthcare Facilities In Qatar to Deal With Potential Mass Casualty Incidents During The FIFA World Cup 2022.Arch Med. 2018;10(1). doi: 10.21767/1989-5216.1000254.

[22] World Health O (2007). Mass casualty management systems: strategies and guidelines for building health sector capacity.

[23] Health Care System Surge Capacity Recognition, Preparedness, and Response: American College of Emergency Physicians (ACEP). ; 2004 [updated 2017; cited 2022 08.12]. Available from: https://www.acep.org/globalassets/new-pdfs/policy-statements/health-care-system-surge-capacity-rec-preparedness-response.pdf. 10.1016/j.annemergmed.2004.10.015 15671992

[24] Tallach R, Einav S, Brohi K, Abayajeewa K, Abback PS, Aylwin C (2022). Learning from terrorist mass casualty incidents: a global survey. Br J Anaesth.

[25] Joslin G, Richardson JW (1975). Staff Recall.

[26] Homier V, Hamad R, Larocque J, Chassé P, Khalil E, Franc JM (2018). A randomized trial comparing telephone tree, text messaging, and instant messaging app for Emergency Department Staff Recall for Disaster Response. Prehosp Disaster Med.

[27] Morris MMSC, Pelley JK, Mitchell MFSH (2016). Using a novel technology for disaster staff notification. J Emerg Med.

[28] Timler D, Bogusiak K, Kasielska-Trojan A, Neskoromna-Jędrzejczak A, Gałązkowski R, Szarpak Ł (2016). Short text messages (SMS) as an additional Tool for Notifying Medical Staff in Case of a hospital Mass Casualty Incident. Disaster Med Public Health Prep.

[29] Pickren A, Harper T (2019). How to implement effective mass notification systems. J Bus Contin Emer Plan.

[30] Von Elm E, Altman DG, Egger M, Pocock SJ, Gøtzsche PC, Vandenbroucke JP (2007). The strengthening the reporting of Observational Studies in Epidemiology (STROBE) statement: guidelines for reporting observational studies. The Lancet.

[31] Barrett D, Heale R (2020). What are Delphi studies?. Evid Based Nurs.

[32] Keeney S, Hasson F, McKenna H (2006). Consulting the oracle: ten lessons from using the Delphi technique in nursing research. J Adv Nurs.

[33] Norwegian population Statistics Norway. ; 2022 [Available from: https://www.ssb.no/en/befolkning/folketall/statistikk/befolkning.

[34] Area of mainland Norway: Statistics Norway. ; 2022 [Available from: https://www.ssb.no/en/natur-og-miljo/areal/statistikk/areal-av-land-og-ferskvatn.

[35] Meld St. 11 (2016–2019). Nasjonal helse- og sykehusplan 2016–2019 (National Health and Hospital Plan 2016–2019) [Electronic]. Oslo: Helse og omsorgsdepartementet (Department of Health); 2015 [42 – 4]. Available from: www.stortinget.no/globalassets/pdf/innstillinger/stortinget/2015-2016/inns-201516-206.pdf.

[36] Allertsen MMT. Legevaktsorganiseringen i Norge, Rapport nummer 3 fra Nasjonalt legevaktsregister 2020 [Electronic]. Norwegian Research Center (NORCE); 2021 [cited 2022 08.12]. Available from: https://norceresearch.brage.unit.no/norceresearch-xmlui/bitstream/handle/11250/2755945/Legevaktorganisering_i_Norge_Rapport_fra_nasjonalt_legevaktregister_2020.pdf.

[37] IBM Corporation (2022). IBM SPSS Statistics for Macintosh. 29.0 ed.

[38] Hsieh HF, Shannon SE (2005). Three approaches to qualitative content analysis. Qual Health Res.

[39] Tavakol M, Dennick R (2011). Making sense of Cronbach’s alpha. Int J Med Educ.

[40] Dae C. Analysis of potential disaster scenarios in Norway: Direktoratet for samfunnsikkerhet og beredskap (Directorate for Civil Protection and Emergency Planning); 2019 [cited 2022. Available from: https://www.dsb.no/globalassets/dokumenter/rapporter/p1808779_aks_2018.cleaned.pdf.

[41] Edwards TD, Young RA, Lowe AF (2007). Caring for a surge of Hurricane Katrina evacuees in primary care clinics. Ann Fam Med.

[42] www.regjeringen.no/contentassets/8087d548c0a04059aa88f416fe19f3cc/no/pdfa/nou199819980009000dddpdfa.pdf.

[43] Desforges JF, Waeckerle JF (1991). Disaster Planning and Response. N Engl J Med.

[44] World Health O. Health emergency and disaster risk management framework. Geneva: World Health Organization; 2019 2019.

[45] Nasjonal veileder for legevakt og legevaktssentral (National guideline for Emergency Primary Health Care Centers). : Helsedirektoratet (The Directorate of Health); 2020 [cited 2022 08.12]. Chapter 7]. Available from: https://www.helsedirektoratet.no/veiledere/legevakt-og-legevaktsentral.

[46] James JJ, Benjamin GC, Burkle FM, Gebbie KM, Kelen G, Subbarao I (2010). Disaster Medicine and Public Health preparedness: A Discipline for All Health Professionals. Disaster Med Public Health Prep.

[47] Developing and Maintaining Emergency Operations Plans., Comprehensive Preparedness Guide (CPG) 101: Federal Emergency Management Agency; 2010 [cited 2022. Version 2,0:[Available from: www.fema.gov/sites/default/files/2020-05/CPG_101_V2_30NOV2010_FINAL_508.pdf.

[48] Dale JN, Morken T, Eliassen KE, Blinkenberg J, Rørtveit G, Hunskaar S (2022). Preparedness and management during the first phase of the COVID-19 outbreak - a survey among emergency primary care services in Norway. BMC Health Serv Res.

[49] World Health Organization. Regional Office for E. Hospital emergency response checklist: an all-hazards tool for hospital administrators and emergency managers. Copenhagen: World Health Organization. Regional Office for Europe. ; 2011 2011. Contract No.: WHO/EURO:2011-4216-43975-61988.

[50] Kuisma M, Hiltunen T, Määttä T, Puolakka J, Boyd J, Nousila-Wiik M (2005). Analysis of multiple casualty incidents–a prospective cohort study. Acta Anaesthesiol Scand.

[51] Kwasinski A, Weaver WW, Chapman PL, Krein PT (2009). Telecommunications Power Plant damage Assessment for Hurricane Katrina– Site Survey and Follow-Up results. IEEE Syst J.

[52] Meng X, Zerfos P, Samanta V, Wong SHY, Lu S. Analysis of the Reliability of a Nationwide Short Message Service2007.1811–9p. doi: 10.1109/INFCOM.2007.211.

[53] World Medical Association Declaration (2013). Of Helsinki: ethical principles for medical research involving human subjects. JAMA.

